# Use of Wearable Transdermal Alcohol Sensors for Monitoring Alcohol Consumption After Detoxification With Contingency Management: Pilot Randomized Feasibility Trial

**DOI:** 10.2196/64664

**Published:** 2025-03-14

**Authors:** Eileen Brobbin, Colin Drummond, Stephen Parkin, Paolo Deluca

**Affiliations:** 1 King's College London London United Kingdom; 2 Department of Public Health Environments and Society at London School of Hygiene and Tropical Medicine King's College London London United Kingdom

**Keywords:** alcohol, alcohol dependence, alcohol monitoring, alcohol treatment, contingency management, transdermal alcohol sensor, transdermal technology, wearable, wearable alcohol biosensor

## Abstract

**Background:**

Wearable transdermal alcohol sensor (TAS) devices generate continuous data on alcohol consumption through the indiscernible sweat vapors on the skin. This continuous alcohol monitoring capability could provide a new method for alcohol services to monitor service users at various stages of their alcohol treatment.

**Objective:**

We aimed to assess the feasibility of using a TAS as part of alcohol treatment with alcohol service users using the device with or without contingency management (CM).

**Methods:**

A feasibility study was conducted of a convenience sample of 29 current service users from 3 South London alcohol services. Participants were randomized into either a control (treatment as usual) or CM group (treatment as usual+CM). We assessed the feasibility of enrollment, participation, device tampering and return, and device wearability and the accuracy of data capture. These data were reported descriptively where appropriate, the groups were compared, and alcohol self-report data were compared to the transdermal alcohol concentration to assess accuracy.

**Results:**

A total of 34 individuals were approached, and 32 (94%) were enrolled and randomized (n=17, 53% to the control group and n=15, 47% to the CM group) over 5 months. In total, 3 participants withdrew (n=2, 67% from the control group and n=1, 33% from the CM group). There was a total of 203 meetings arranged (29 participants × 7 meetings), and 185 (91.1%) were attended. Only 1 of the 29 participants (3%) admitted to turning the TAS off to avoid monitoring. There were some issues with the TAS not functioning properly and not being able to be cleaned. Removals were recorded, but the definition of TAS removal may need to be improved for future trials. There was a high TAS return rate (28/29, 97% of the participants returned the TAS). Secondary outcomes suggest that the BACtrack Skyn remains an accurate tool to monitor alcohol consumption compared to self-report data and that it is acceptable to wearers over 2 weeks, with many participants (27/28, 96%) answering that they would wear it again and for longer but that the CM procedure could be made clearer.

**Conclusions:**

The delivery of CM via a TAS was feasible in this study, but recommendations for a future larger trial include that the study design should be changed to provide an operationalized rather than manual method of checking whether TAS data meet CM criteria. This would reduce researcher burden and researcher and participant time. Current recruitment and research meeting design seem suitable for a future larger trial.

**Trial Registration:**

International Standard Randomised Controlled Trial Number (ISRCTN) ISRCTN46845361; https://www.isrctn.com/ISRCTN46845361

## Introduction

### Background

Various wearable transdermal alcohol sensor (TAS) devices have been developed. These devices measure alcohol consumption from alcohol vapors in the skin via sweat, known as transdermal alcohol concentration (TAC), and can record at automated regular intervals. One potential use of TASs is as a tool for objective alcohol measurement in clinical alcohol treatment.

TASs could be used in specialist community alcohol services for monitoring alcohol consumption before detoxification to determine drinking levels and patterns, during detoxification, or after detoxification to be combined with sessions with key workers and other psychosocial or medication treatment. The recorded alcohol data could be used to consider triggers and provide evidence of abstinence or alcohol reduction and proof of engagement, which may be useful as evidence for funded treatment, or TASs could be used to implement contingency management (CM) for alcohol reduction. However, before TASs can be implemented into treatment, their accuracy, acceptability, and feasibility in this setting must be validated.

There have been several studies that have investigated the various TAS device brands on TAC data accuracy compared to self-report, blood alcohol concentration, and breath alcohol content data [1-17]. Most of this previous research was conducted with healthy adult volunteers [18,19]. Various brands of TASs have been developed and are in differing stages of validation and research. There have only been a few studies conducted with a specific focus on exploring TAS acceptability and feasibility measures [12,20-26] and even fewer specifically on TAS implementation of CM rewards [1,2,26-31]. While most of the TAS research reports on the SCRAM monitor, there is a growing number of individuals using a newer generation of TASs, the BACtrack Skyn [7,12-14,32-42].

CM has been evidenced as an effective treatment for substance use. It is an established treatment recommended by the National Institute for Health and Care Excellence [43] and has proven to be effective for a range of substance use treatments [44-47]. Although initially developed to be used with alcohol use disorder (AUD) treatment, it has had limited use in this area [48,49]. This is due to the nature of alcohol metabolism and its short detectability within the body [50,51].

Currently used methods for alcohol measurement include breath, blood, and urine tests, which have a relatively short time frame to detect alcohol [52]. Thus, to accurately implement CM in alcohol treatment, the individual would require frequent, multiple breath, blood, or urine tests daily to prove abstinence or alcohol reduction and accurately achieve CM rewards [47,49,53,54]. This is not always feasible with staff time and resources and increases the burden on both service users and staff. The portable mobile phone–linked breathalyzer has also tried to address this limitation and appears to work well in tracking alcohol treatment progress [55-58]. However, it still requires repeated daily compliance from the individual completing each breathalyzer test.

The development of TASs has the potential to address CM implementation with alcohol use and can provide a low-burden, low-intensity solution [1,2,26-31]. Previous literature has started to explore how TASs can implement CM [1,2,26-31]. These studies have found the SCRAM monitor successful in implementing the CM procedure and found that the CM intervention was able to significantly reduce alcohol consumption [1,2,27,29]. Of these 8 studies, none recruited alcohol-dependent participants. In total, 2 involved recent driving while intoxicated offenders with differing criteria on alcohol consumption, 1 with an Alcohol Use Disorders Identification Test score of ≥4 [28] and the other with an Alcohol Use Disorders Identification Test score of ≥8 [30]; 2 recruited HIV-diagnosed individuals with higher levels of alcohol consumption [26,31]; and the other 4 classified participants as risky or heavy drinkers with varying ways to define this [1,2,27,29]. The length of TAS wear periods and CM length ranged from 1 to 4 months, and all studies were conducted in the United States and used the SCRAM monitor.

This study followed a previous study that used the Skyn with individuals in alcohol treatment wearing the device for 1 week and interviewed staff on their thoughts [36,59,60]. From this previous study, we found that most of the individuals were willing to wear the device for longer and that staff want patients wearing the device for longer than a week for it to be used in alcohol treatment. Therefore, for this study, we extended the wear time for 2 weeks and added the CM component. The findings of this study will contribute to the knowledge on TAS feasibility in alcohol research and providing CM in alcohol services in South London.

### Objectives

This study aimed to explore the feasibility, strengths, and limitations of using a TAS to monitor alcohol consumption in individuals in treatment for AUD with or without CM to promote abstinence or low-level alcohol consumption.

## Methods

This was a randomized controlled trial with a 1:1 allocation ratio to the control and CM groups.

### Patient and Public Involvement and Staff Consultation

To ensure that the recruitment, study design, and outcomes were appropriate, when designing this study, we conducted monthly patient and public involvement groups and staff consultation for 6 months. Service users and staff from 3 South London alcohol services were invited to attend. There were 2 meetings held each month, one for service users (in person) and one for staff (on Microsoft Teams). In these groups, we discussed study aspects and the participants’ thoughts on potential challenges.

### Setting

In total, 3 drug and alcohol services were recruited from South London and Maudsley National Health Service Foundation Trust. The 3 services were the Pier Road Project (Erith), Wandsworth Community Drug and Alcohol Service (Wandsworth and Richmond), and the Assertive Alcohol Outreach Team (Camberwell). All services were willing to be involved in this study and had recruitment occur at their facilities. At each service, after referral, patients typically completed a community detoxification, were prescribed any medication if appropriate, and started to attend group meetings and regular one-to-one meetings with their key worker. All participants that were recruited for this study were currently attending groups and one-to-one sessions.

### Participants and Sample Size

Participants were referred by service staff and by the researcher attending patient groups to discuss the study. One of the aims of this trial was to investigate the feasibility of conducting a larger trial. Therefore, this study conducted no formal statistical sample size calculation but aimed to recruit 30 participants (15 in each arm).

The inclusion criteria were (1) reception of alcohol treatment for an AUD in one of the participating South London alcohol services, (2) age of ≥18 years, (3) ability to speak English competently, (4) ability to meet throughout the study period, (5) no current participation in any other research trials, and (6) willingness to provide informed consent to participate. Exclusion criteria were (1) current use of any illegal or addictive substances (excluding cannabis), (2) age of <18 years, and (3) inability to speak English without a translator.

The study inclusion criteria were intentionally kept broad, enabling individuals receiving any treatment for an AUD (provided that they met the other criteria) to participate.

### Randomization

Participants were randomized via dedicated software and sealed envelopes by an independent statistician. The team member recruiting participants (EB) was not aware of the randomization allocation sequence until the sealed numbered envelope was opened at each participant’s first research meeting. EB enrolled all participants and assigned participants to their allocated group. The researcher and participants were not blind to the allocation; both were aware of whether they were receiving CM rewards.

### Procedure

At enrollment, participants were randomized into one of two groups: (1) treatment as usual+wearing a TAS (control group) or (2) treatment as usual+wearing a TAS+CM for low or no alcohol consumption as measured using the TAS (CM group).

Each participant had 7 research meetings arranged across 15 days. At the first meeting, the participant was trained in using the TAS and provided with a quick leaflet guide to take home with them, had the study protocol explained, and was randomized into a group. Meetings 2 to 6 were for TAS data download and timeline followback (TLFB) completion. At the final meeting, meeting 7, participants also had TAS data downloaded and completed the TLFB as well as a postwear survey. This procedure is shown in Table 1.

**Table 1:** Study procedure—an example of the research meetings if the first meeting happened on a Monday.

		Meeting description
**Week 1**		
	Monday	Meeting 1—talk participants through the study and train them in using the TAS^a^
	Wednesday	Meeting 2—TAS data download and TLFB^b^ (+CM^c^)
	Friday	Meeting 3—TAS data download and TLFB (+CM)
**Week 2**		
	Monday	Meeting 4—TAS data download and TLFB (+CM)
	Wednesday	Meeting 5—TAS data download and TLFB (+CM)
	Friday	Meeting 6—TAS data download and TLFB (+CM)
**Week 3**		
	Monday	Meeting 7—TAS data download, TLFB (+CM), and postwear survey

^a^TAS: transdermal alcohol sensor.

^b^TLFB: timeline followback.

^c^CM: contingency management.

If the participant was randomized into the CM group, in addition to the aforementioned steps, at each meeting (meetings 2-7), the TAS data were checked to confirm whether the participant had been abstinent or consumed an amount of alcohol below our set threshold and whether they had been wearing the TAS. If they met these 2 criteria, they were then rewarded with the corresponding amount for the days of abstinence or low level of drinking.

The reason for the research meeting design was for regular TAS data download to ensure that the TAS did not start overwriting data. At the time of this study, the Skyn could store up to 72 hours before overwriting data. Participants also completed a TLFB with the researcher at every meeting, so they only had to recall alcohol consumption for the previous 2 to 3 days [61].

### CM Intervention

Participants who were randomized to the CM group could also earn rewards by being abstinent or for low drinking as measured using the TAS and wearing the TAS consistently (we specified that removal of no more than 60 minutes per day was allowed for a shower or bath). The CM reward was a £5 (US $6.25) voucher for each day that the target behavior occurred. There were also bonuses for consecutive days of the target behavior occurring. At each meeting (meetings 2-7), the researcher checked their TAC data and provided any earned CM rewards since the previous meeting. If the participant met the target behavior every single day for the study period, there was an additional bonus given at the end (£35 [US $43.75]). Therefore, a total of £180 (US $225.02) over the study period could be given in CM vouchers. If there was a day in which this behavior did not occur, then participants received no CM for that day and were not eligible for that bonus. The CM plan is shown in Table 2.

**Table 2:** Contingency management (CM) plan—an example of the CM plan for a Monday start. Participants were ineligible for CM if the device was removed (skin temperature of <30 °C for >1 hour). A 1-time 1-hour removal was allowed per day.

Day number	Day	CM for 1-day abstinence (£5 [US $6.25] per day)^a^	CM bonus for consecutive-day abstinence (£5 [US $6.25] per day)^b^	CM bonus for 14 consecutive days of abstinence (£35 [US $43.75] for 14 days)^c^
1	Monday	£5 (US $6.25)	—^d^	—
2	Tuesday	£5 (US $6.25)	—	—
3	Wednesday	£5 (US $6.25)	£10 (US $12.50; second meeting)	—
4	Thursday	£5 (US $6.25)	—	—
5	Friday	£5 (US $6.25)	£10 (US $12.50; third meeting)	—
6	Saturday	£5 (US $6.25)	—	—
7	Sunday	£5 (US $6.25)	—	—
8	Monday	£5 (US $6.25)	£15 (US $18.75; fourth meeting)	—
9	Tuesday	£5 (US $6.25)	—	—
10	Wednesday	£5 (US $6.25)	£10 (US $12.50; fifth meeting)	—
11	Thursday	£5 (US $6.25)	—	—
12	Friday	£5 (US $6.25)	£10 (US $12.50; sixth meeting)	—
13	Saturday	£5 (US $6.25)	—	—
14	Sunday	£5 (US $6.25)	—	—
15	Monday	£5 (US $6.25)	£15 (US $18.75; seventh meeting)	£35 (US $43.75; seventh meeting)

^a^Total of £75 (US $93.76).

^b^Total of £70 (US $87.51).

^c^Total of £35 (US $43.75).

^d^Not applicable.

### Measures

#### BACtrack Skyn

The TAS used in this study was the BACtrack Skyn (model T15). It was worn on the participants’ preferred wrist, but they could change which wrist they wore it on during the study period if that was comfortable for them. The Skyn continuously measured the participant’s TAC while being worn, as well as skin temperature (°C). Output was analyzed at 1-minute intervals. The participants could remove the TAS at any time if they did not wish to wear it and were required to remove it for bathing as it is not waterproof. The CM group were told that they could remove it once a day for up to 60 consecutive minutes to bathe and still be eligible for their CM reward. If it was removed for longer than an hour, they would no longer be able to receive the CM reward. If they wore the TAS according to this and the TAC did not increase above our set threshold of 115.660 µg/L, they were eligible for the CM reward for that day. Our set threshold was based on previous work by the research team [35].

#### TLFB Method

A TLFB was completed at meetings 2 to 7 to assess self-reported alcohol consumption and compare it against TAC. The TLFB is a calendar-based measure to record self-reported substance use. A day was considered from midnight to 11:59 PM.

#### Postwear Surveys

Participants completed a postwear survey on their experience of wearing the Skyn at their last meeting. This survey was adapted from the work by Alessi et al [20]. If participants were randomized to the CM group, they also completed a survey on their CM experience. This survey was adapted from the work by Miguel et al [62].

### Outcomes

#### Feasibility of the Trial

This primary outcome was the feasibility of this study design. Feasibility was defined by enrollment, participation, device tampering, removals, adjustments, malfunction rates, and the number of TASs returned.

#### Enrollment

Participants who were identified, approached, eligible, and enrolled were recorded. Participants who were approached but did not wish to participate were asked about their main reason for this. Participant safety was recorded via adverse events for each participant. The number of participants enrolled on each service was also recorded.

#### Participation

The number of attended meetings by enrolled participants, withdrawal rate, compliance with wearing the TAS, and reasons for incomplete participant data (nonattendance, TAS data overwriting, and TAS technical faults) were recorded. Participants were asked what their main reason was for nonattendance or for not complying with TAS wear.

#### Removals

TAS removals were defined as a skin temperature of <30 °C for >2 minutes. Removals were recorded for each participant.

#### Tampering

Participants were asked about tampering with the TAS if there was no clear reason for missing TAC data in the output.

#### Malfunction

TAS errors, missing data due to a technical fault, and charging or syncing issues were recorded, as well as how much data were missing for these reasons.

#### TAS Return

The number of Skyn devices that were returned to the research team at the end of each participant’s study period was recorded.

#### CM Delivery

The feasibility of TASs to measure CM target behavior was a combination of the factors mentioned previously, including compliance, removals, TAS tampering, malfunction, and accuracy.

#### Secondary Outcomes

##### Acceptability

This was measured using the postwear survey.

##### Accuracy

Accuracy outcomes were measured using the TLFB and TAC data. The TLFB was self-reported [63] and was used to determine alcohol-drinking days and how many units were consumed (in the United Kingdom, 8 g or 10 mL of pure ethanol=1 unit). The TAS (BACtrack Skyn) continuously measured TAC and skin temperature (°C), and from this, Skyn removal and then reported drinking days based on the TAC (µg/L) were determined. The TLFB and TAC data were analyzed to determine TAS accuracy compared to self-report data.

To note, the alcohol-drinking day defined using TAC is different to the CM intervention criteria, which allowed for a low amount of drinking and had a higher TAC criterion of 115.660 µg/L.

### Data Handling

There is currently no guidance from BACtrack to determine drinking event criteria. Courtney et al [34] described their procedure for processing Skyn output to identify drinking episodes, and we used these guidelines when processing participant data. We replaced any negative values recorded with 0 in the data output. Missing data were classified as any minutes not reported. Removed data were those reporting a temperature of <30 °C for >2 minutes.

An alcohol event was based on TAC greater than a specific value (µg/L) for more than a set number of minutes. These criteria were chosen due to previous work carried out by this research team [35,59].

### Analysis

Descriptive statistics were reported for baseline and demographic variables. All statistical analyses were conducted using SPSS (version 28; IBM Corp).

The feasibility outcomes reported included enrollment and recruitment rate, participant attendance, response and compliance, removals, TAS tampering and error, and TAS return. Appropriate summary statistics were reported, and independent-sample 2-tailed *t* tests were used to compare means between the control and CM groups when appropriate.

Summary statistics were reported for the secondary outcomes to be explored for a possible future larger trial. The postwear survey answers were reported. TAS accuracy was determined by analyzing the TAS data compared to self-report TLFB. The analysis focused on the sensitivity, specificity, positive predictive value, negative predictive value, and percentage accuracy in classification of TAC compared to TLFB as the gold standard. Recorded drinking and abstinent days were assessed using Spearman rank correlations. Sensitivity in detecting alcohol events and specificity in classifying an alcohol-drinking day versus a non–alcohol-drinking day were assessed using different TAC criteria: TAS 15 (TAC>15 µg/L for >15 min), TAS 60 (TAC>15 µg/L for >60 min), and TAS 90 (TAC>15 µg/L for >90 min).

### Ethical Considerations

This study was approved by the Cornwall and Plymouth Research Ethics Committee (reference: 23/SW/0066) and registered on the Open Science Framework and International Standard Randomised Controlled Trial Number (reference: ISRCTN46845361). Informed written consent was obtained from all participants and their data were anonymized. All participants received a £5 (US $6.25) voucher at every meeting they attended (total for all 7 meetings=£35 [US $43.75]), a voucher for the return of the TAS on the last meeting (£10 [US $12.50]), and reimbursement for travel costs to each meeting. Therefore, each participant could have received up to £45 (US $56.25) for their participation plus travel costs.

## Results

### Participants

**Figure 1:**
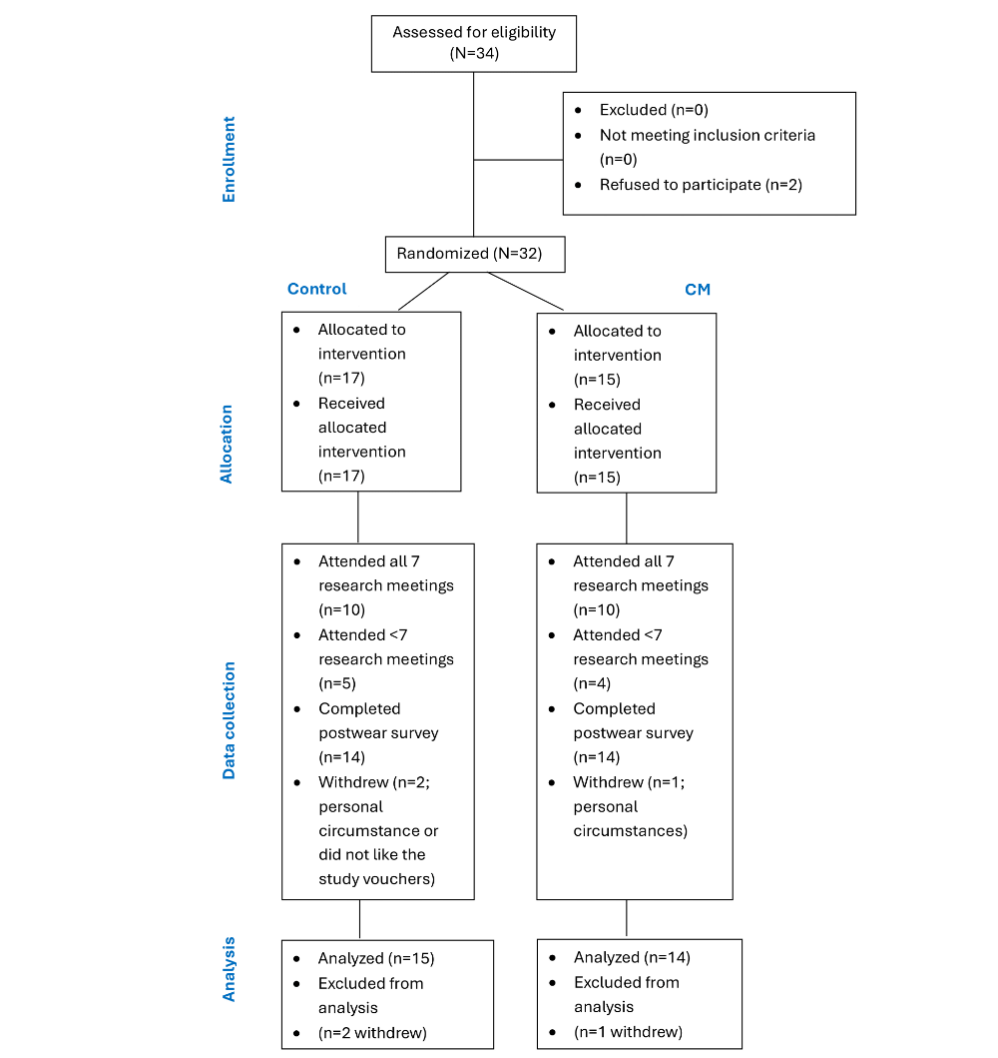
Flowchart of participant recruitment and retention. CM: contingency management.

A total of 32 healthy adult participants (n=10, 31% female and n=22, 69% male) enrolled, and a total of 29 (91%) completed the study. In total, 3 participants withdrew (n=2, 67% from the control group and n=1, 33% from the CM group). Of these 3 withdrawals, 2 (67%) happened during the study period (P8 [control group] on day 3 and P28 [CM group] on day 12), and 1 (33%) occurred at the end of the first meeting (control group) due to the specific type of voucher, which could not be used at the participant’s local supermarket. P8 and P28 withdrew because of personal circumstances changing. Therefore, a total of 29 participants were included in the analysis (n=10, 34% female and n=19, 66% male; Figure 1). Table 3 shows the characteristics of the 29 participants included in the analysis split by group (control vs CM).

**Table 3:** Differences in group characteristics.

Characteristic			Control group (n=15)		CM^a^ group (n=14)		Group intervention difference	
**Sex^b^, n (%)**								*χ*^2^_1_=0.4; *P*=.52
	Female	6 (40)		4 (28.6)				
	Male	9 (60)		10 (71.4)				
Age (y), mean (SD; range)			44.4 (13.78; 30-75)		51.5 (9.77; 35-60)		*P*=.12	
BMI (kg/m^2^), mean (SD; range)^c^			27.3 (6.24; 20.8-42.3)		28.7 (6.35; 22.2-42.5)		*P*=.58	
Height (cm), mean (SD; range)			168.17 (6.40; 162-177)		171.29 (7.49; 162-185)		*P*=.24	
Weight (kg), mean (SD; range)			77.2 (17.16; 53-112.5)		84.63 (19.75; 59-126.33)		*P*=.31	
**Ethnicity^b^, n (%)**								*χ*^2^_16_=43.3; *P*<.001
	Black African	3 (20)		0 (0)				
	Black British	2 (13.3)		0 (0)				
	Caribbean	0 (0)		1 (7.1)				
	Hispanic	1 (6.7)		0 (0)				
	White British	7 (46.7)		9 (64.3)				
	White Irish	1 (6.7)		3 (21.4)				
	White Polish	0 (0)		1 (7.1)				
	White Russian	1 (6.7)		0 (0)				
Units of alcohol consumed, mean (SD; range)			117.14 (105.83; 0-316.69)		53.33 (93.21; 0-294.69)		*P*=.14	
Days in which alcohol was consumed (TLFB^d^), mean (SD)			8.14 (6.44)^e^		4.50 (6.62)^f^		*P*=.08	
Average units consumed over days of alcohol consumption, mean (SD)			117.14^g^ (105.83)		53.33^h^ (93.21)		—^i^	
TAS^j^ removals (min), n (%)			60,879 (20.8)^k^		30,103 (11)^l^		*P*=.08	
TAS missing data (min), n (%)			28,213 (9.6)^k^		28,591 (10.4)^l^		*P*=.80	
Meetings attended, n (%)			94 (89.5)^m^		91 (92.9)^n^		*P*=.57	
Participants who completed the postwear survey, n (%)			14 (93.3)		14 (100)		*P*=.34	
Participants who returned the TAS, n (%)			14 (93.3)		14 (100)		*P*=.34	

^a^CM: contingency management.

^b^Ethnicity is represented as the frequency of individuals in each group identifying as Black African, Black British, Caribbean, Hispanic, White British, White Irish, White Polish, and White Russian and was compared across sexes using a chi-square analysis.

^c^One female and one male participant requested not to be weighed, so BMI could not be calculated (both in the control group).

^d^TLFB: timeline followback.

^e^Total of 114 days.

^f^Total of 63 days.

^g^1640.01 units over 114 days.

^h^746.67 units over 63 days.

^i^No group comparison was possible.

^j^TAS: transdermal alcohol sensor.

^k^n=293,186.

^l^n=274,218.

^m^n=105.

^n^n=98.

### Feasibility of the Trial

#### Enrollment

A total of 34 potentially eligible participants were approached. Only 6% (2/34) who were identified by staff declined to participate after discussing the study with the researcher. Their reasons were (1) that they did not like wearing a watch so did not think that they would like to wear a TAS and (2) that they were very busy and could not commit to the regular meetings. No adverse events occurred. The services involved were also willing to help with recruitment, and all recruited at least 4 participants over the 5-month recruitment period.

#### Participation

It was feasible to enroll more than our target sample size (30 participants) within 5 months. The first participant was enrolled on July 5, 2023, and the final participant (N=32) was enrolled on December 6, 2023. These data could be used to inform the sample size power calculation for a future larger trial.

Among the 29 participants who completed the study period and did not withdraw, a total of 203 meetings were arranged (29 × 7 visits). Of these 203 meetings, 185 (91.1%) were attended. Reasons for nonattendance included illness, their partner being ill, being double booked with another health appointment, a broken phone so the researcher could not be contacted, and hospitalization. The research team decided to book research meetings one at a time, so at meeting 1, meeting 2 was agreed upon, and so on. The day before each meeting, a reminder SMS text message was sent out with the time and date and asking the participants to let the research team know whether this needed to be changed. This design seems feasible to maintain a high attendance rate.

Only 1 of the 29 participants (3%) spoke to the research team about removing their TAS early and removed it at the sixth meeting (2 days early) because they did not want to wear it anymore as they felt that “it is messing with my head” (P22).

Among the 3 services involved, the Assertive Alcohol Outreach Team recruited 53% (17/32) of the participants, the Pier Road Project recruited 34% (11/32) of the participants, and Wandsworth Community Drug and Alcohol Service recruited 12% (4/32) of the participants.

#### Removals

We defined TAS removal as >2 minutes during which the skin temperature was of <30 °C. We reminded participants to wear the TAS as much as possible, including while asleep. However, the TAS is removable by the wearer, and they could choose not to wear it if they wanted to and must remove it for water activities (bathing and swimming). Of the total minutes collected from the 29 participants (464,324 minutes of data, approximately 322 days), there was a total of 19.59% (90,982/464,324) of minutes of removals (approximately 63 days).

The control group had a total of 20.21% (60,879/301,179) of minutes of removals. The CM group had a total of 10.79% (30,103/278,862) of minutes of removals (t_27_=1.843; *P*=.08; *d=*0.685).

#### Tampering

One participant (P1) admitted to turning the Skyn off while wearing it when they did not want to be monitored. No other evidence or reports of tampering from any other participant was recorded. This participant had 65.65% (13,173/20,067) of minutes of their participation time successfully recorded and 34.35% (6893/20,067) of missing data. Of these missing data, approximately half could be due to not attending meetings and data being overwritten (3386/6893, 49.12%). However, all other meetings were attended, so the 50.88% (3507/6893) of minutes of missing data could be due to the TAS being turned off or technology error and additional data overwriting (approximately 2.5 days’ worth of data).

#### Malfunction

A total of 16 Skyns were ordered in phases during the study period. The first order of 6 Skyns had 2 issues: one Skyn had trouble pairing for the first 2 weeks, and another would not charge or pair with any research iPhone. BACtrack sent 3 Skyn replacements, and 2 of these would not pair with a research iPhone at first (they did 1 month later). We ordered a second batch of 7 Skyns halfway through data collection, and there were no issues with any devices from this order. BACtrack were unsure about the reasons for the issue with pairing and charging. Other issues experienced included syncing taking >40 minutes on occasions (the usual time is approximately 5 minutes). This was most likely due to poor connection or Wi-Fi.

Apart from 1 Skyn that arrived and could not be charged, there were no charging issues. The Skyn website states that the battery lasts for approximately 10 days; however, we found that it lasted for >2 weeks. Participants were provided with a fully charged Skyn at the first meeting. There were 2 participants whose battery decreased to a low level within their 2-week study period, so the warning red light started flashing. As the TASs were typically able to retain the charge for the entire study period, we did not provide the charging cable to participants. If the TASs will be used for >2 weeks in a future study, either the charging cable will need to be provided or there will need to be planned charging time with the researchers.

**Figure 2:**
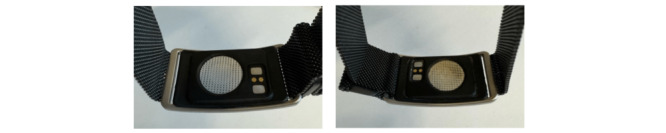
These 2 photos of the BACtrack Skyn sensor show a brand-new, unworn Skyn (left) and a worn Skyn (right).

There was evidence of Skyn degradation after 2 months, which is partly why it was decided to order another batch of Skyns halfway through data collection [33]. Another reason for this order was their state of cleanliness (Figure 2). BACtrack do provide guidelines on how to clean the sensor; however, after following these instructions, the sensor could not be cleaned well.

#### TAS Return

All but 1 Skyn were returned. This was due to the participant disengaging from the service and not being contactable by the researcher or key worker. One other participant did not attend their last research meeting, but they arranged an additional meeting to return the TAS.

#### CM and Intervention Delivery

CM rewards were delivered if 2 criteria were met: (1) the TAC for the day (midnight-11:59 PM) had a peak of <115.660 µg/L and (2) there were no removals lasting >60 minutes (temperature of <30 °C).

The TAS data were downloaded at each meeting (meetings 2 to 7) and checked, and any CM vouchers were provided for the previous 2 to 3 days. This was considered the best way to implement CM as close to those days in which the behavior occurred as possible. Table 4 details the amount of CM rewards earned by each participant and any reason why CM rewards were not earned.

**Table 4:** Contingency management (CM) rewards earned by participants in the CM group.

Participant ID	Days in which CM rewards were earned^a^	Total amount earned^b^	Days in which CM rewards were not earned^c^	Reason why CM rewards were not earned^d^
P4	0	£0	15	15 days=TAC^e^ over the limit
P5	13	£105 (US $131.26)	2	1 day=TAC over the limit; 1 day=removal for >60 min
P6	12	£95 (US $118.76)	3	1 day=TAC over the limit; 2 days=removal for >60 min
P7	10	£85 (US $106.26)	5	1 day=TAC over the limit; 4 days=removal for >60 min
P10	12	£90 (US $112.51)	3	1 day=TAC over the limit; 2 days=removal for >60 min
P11	0	£0	15	15 days=TAC over the limit
P16	2	£10 (US $12.50)	13	6 days=removal for >60 min; 7 days=DNA^f^ appointment and data overwritten
P17	5	£25 (US $31.25)	10	6 days=TAC over the limit; 2 days=removal for >60 min; 2 days=DNA appointment and data overwritten
P18	9	£55 (US $68.75)	6	6 days=removal for >60 min
P19	9	£75 (US $93.76)	6	3 days=removal for >60 min; 3 days=DNA appointment and data overwritten
P21	13	£110 (US $137.51)	2	2 days=removal for >60 min
P22	1	£5 (US $6.25)	11	9 days=TAC over the limit; 2 days=removal for >60 min; handed TAS^g^ back 2 days early
P25	3	£15 (US $18.75)	12	2 days=TAC over the limit; 10 days=removal for >60 min
P29	11	£85 (US $106.26)	4	4 days=removal for >60 min

^a^Total: 48.3% (100/207) of days.

^b^Total: £755 (US $943.82).

^c^Total: 51.7% (107/207) of days.

^d^Total: 24.6% (51/207) of days with transdermal alcohol concentration over the limit, 21.3% (44/207) of days of removals, and 5.8% (12/207) of days of data overwritten.

^e^TAC: transdermal alcohol concentration.

^f^DNA: did not attend.

^g^TAS: transdermal alcohol sensor.

Control group participants attended 89.5% (94/105) of the meetings, and the CM group participants attended 92.9% (91/98) of the meetings (t_27_=−0.573; *P*=.57; *d=*−0.213). The control group self-reported 48.6% (107/220) of abstinent days, and the CM group self-reported 70% (147/210) of abstinent days (t_27_=−1.403; *P*=.17; *d=*−0.522). The proportion of days in which the Skyn data reported <1 hour of removals was 15.9% (35/220) for the control group and 24.8% (52/210) for the CM group (t_27_=−1.326; *P*=.20; *d=*−0.493). The difference in the amount of units consumed between groups was not statistically significant (control: mean 109.33, SD 27.47; CM: mean 53.33, SD 24.91; t_27_=1.503; *P*=.14; *d*=0.559).

#### Acceptability and Postwear Survey

A total of 97% (28/29) of the participants completed the postwear survey. One participant did not complete it because they did not attend their final research meeting and became unreachable. When asked about physical comfort, most participants (11/28, 39%) rated it to be quite to very comfortable, with the average score being 8.57/10 (SD 0.71; 10=very comfortable). Social comfort (how they felt wearing it in public) was also rated highly, with an average score of 9.63/10 (SD 0.00; 10=very socially comfortable). When asked how often they noticed the Skyn on their wrist, only 11% (3/28) of the participants said that they never noticed it when wearing it. Most (18/28, 64%) noticed it once or twice a day to every hour, but 25% (7/28) did report noticing it several times per hour. When asked to rate its interference with various activities (exercise, mood, normal work, enjoyment of life, ability to concentrate, social life, and clothing choices), these were all rated with an average score of <2 (1=no interference at all; general activity: 1.75, SD 0.00; exercise: 1.07, SD 0.00; mood: 1.11 SD 0.00; work: 1.07, SD 0.00; enjoyment of life: 1.21, SD 0.00, ability to concentrate: 1.43, SD 0.00; social life: 1, SD 0.00; choice of clothing: 1.24, SD 0.00). The only activity that ranked higher was sleeping, with an average score of 2.71 (SD 2.83; 1=no interference at all).

A total of 32% (9/28) of the participants reported experiencing a mark on their skin from the TAS, with the other 68% (19/28) reporting never experiencing a mark or side effect. These side effects included itching (mean 1.95, SD 0.00), sweating (mean 1.88, SD 0.00), soreness (mean 1.07, SD 0.00), and irritation (mean 1.50, SD 0.00), with the scale being from 1=not noticeable to 10=unbearable. Participants were asked whether they would continue to wear the TAS for longer than the 2-week study period, and 96% (27/28) reported *yes*, and only 4% (1/28) reported *no*.

The following statements—“the device is too uncomfortable to wear for any longer,” “I wouldn’t want to wear it any longer because I am embarrassed,” and “I want to wear short sleeves but won’t while wearing the device”—were answered by all participants with “not true.” Statements asking about changing their clothing choices, wanting to remove the TAS, and not liking the regular download visits all had an average score of 0.25 (SD 0.00 for all these statements) (on a scale of 0-4 where 0=*not true* and 4=*very true*). Only 4% (1/28) of the participants stated that they were tired of explaining the device to people. In total, 4% (1/28) stated that they were “ready to stop wearing the device because I am just ready to be done,” and another participant (1/28, 4%) said that the “financial compensation for wearing the device any longer than this would not be worth it.” A total of 7% (2/28) of the participants said that it was true that “I would not continue wearing the device because I do not like having to do the downloads at a specific time.”

When asked about their drinking, 11% (3/28) self-reported that they were able to completely reduce their drinking, 25% (7/28) reported that they reduced it quite a bit, 14% (4/28) reported that they reduced it somewhat, 7% (2/28) reported that they tried but did not reduce it, and 11% (3/28) reported that they did not reduce or try to reduce it. In total, 32% (9/28) of the participants were abstinent throughout their study period.

Many participants (12/28, 43%) liked the in-person vouchers that were given at each meeting; however, 7% (2/28) would have preferred an e-voucher, 21% (6/28) would have preferred a bank transfer, and 18% (5/28) would be happy with any of these options (in-person voucher, e-voucher, or bank transfer). In total, 7% (2/28) reported preferring only an e-voucher or bank transfer, and 4% (1/28) preferred an in-person voucher or a bank transfer. Finally, participants were asked about the ease of meeting the researcher for the meetings, and 89% (25/28) reported that it was *not at all difficult*, whereas the other 11% (3/28) reported that it was *a little bit difficult*.

#### CM Survey

The 48% (14/29) of participants who were allocated the CM group completed another survey specifically on their experience of the CM rewards. All participants in the CM group (14/14, 100%) completed this survey. When asked about how easy it was to understand the CM reward procedure, most (9/14, 64%) said that it was very easy, but 21% (3/14) rated it as very difficult. When asked whether the CM had any effect on their response to treatment, half (7/14, 50%) said that it helped them either a lot or a little, 43% (6/14) said that it did not make a difference, and 7% (1/14) said that it had a negative impact. When asked whether they liked the CM rewards, all (14/14, 100%) said that they liked them either a lot or a little. When asked when CM rewards would help other people who seek substance treatment for alcohol dependence, 86% (12/14) said that it would help them a lot or a little, and 14% (2/14) said that it would depend on the person.

#### Accuracy

Participants wore the TAS for a cumulative total of 580,040 minutes. A total of 80.05% (464,324/580,040) of minutes of this participation was successfully recorded by the TAS. Of these 464,324 minutes of recorded data, there was a total of 90,982 (19.59%) minutes that suggested removal, a total of 56,858 (12.25%) minutes that were missing for unknown reasons, and a total of 58,986 (12.7%) minutes that were missing due to being overwritten as a result of not attending appointments. The Skyn is not waterproof, so participants were required to remove it for showering, baths, and any other water activities (eg, swimming). No fit adjustments were required.

The TAS recorded data for a total of 388 days. Of these 388 days, 337 (86.9%) had >60 minutes of removals or missing data. There was a total of 68.6% (266/388) of days in which there were >300 minutes of removed or missing data (not necessarily a consecutive 5-hour period).

#### TLFB Versus Skyn TAC

##### Overview

TLFB drinking days were recorded via the participant reporting any alcohol consumed. TAS drinking days were recorded according to the TAC criteria. We considered 3 different criteria for detecting alcohol-drinking days: TAC of >15 µg/L for >15 minutes, TAC of >15 µg/L for >60 minutes, and TAC of >15 µg/L for >90 minutes. The agreement between the TLFB and these criteria is reported in Table 5.

**Table 5:** Timeline followback (TLFB)– and transdermal alcohol sensor (TAS)–reported alcohol-drinking and nondrinking days.

Criteria			Alcohol-drinking days detected		Alcohol-drinking days in agreement (n=388), n (%)			Non–alcohol-drinking days in agreement (n=388), n (%)			Days reported by the TAS but not by TLFB as alcohol-drinking days (n=388), n (%)			Days reported by TLFB but not by the TAS as alcohol-drinking days (n=388), n (%)	
**TAC^a^ >15 µg/L for >15 min**						164 (42.3)			145 (37.4)			65 (16.8)			14 (3.6)
	TLFB	185													
	TAS	227													
**TAC>15 µg/L for >60 min**						146 (37.6)			171 (44.1)			39 (10.1)			32 (8.2)
	TAS	185													
	TLFB	185													
**TAC>15 µg/L for >90 min**						139 (35.8)			179 (46.1)			31 (8)			39 (10.1)
	TAS	185													
	TLFB	170													

^a^TAC: transdermal alcohol concentration.

##### Criteria for a Drinking Event of TAC>15 µg/L for >15 Minutes

The TLFB and TAS agreed on 42.3% (164/388) of days as alcohol-drinking days and on 37.4% (145/388) of days as abstinent days. There were 3.6% (14/388) of days in which the TLFB reported an alcohol-drinking day and the TAS did not and 16.8% (65/388) of days in which the TAS reported an alcohol-drinking day and the TLFB did not.

When splitting the groups and conducting a correlation between TAS- and TLFB-reported drinking days, both were positively significantly correlated, but the CM group had a stronger correlation effect (control group: *r*_15_=0.625 and *P*=.01; CM group: *r*_14_=0.836 and *P*<.001).

##### Criteria for a Drinking Event of TAC>15 µg/L for >60 Minutes

The TLFB and TAS agreed on 37.6% (146/388) of days as alcohol-drinking days and on 44.1% (171/388) of days as abstinent days. There were 8.2% (32/388) of days in which the TLFB reported an alcohol-drinking day and the TAS did not and 10.1% (39/388) of days in which the TAS reported an alcohol-drinking day and the TLFB did not.

When splitting the groups and conducting a correlation between TAS- and TLFB-reported drinking days, both were positively significantly correlated, but the CM group had a stronger correlation effect (control group: *r*_15_=0.764 and *P*<.001; CM group: *r*_14_=0.895 and *P*<.001).

##### Criteria for a Drinking Event of TAC>15 µg/L for >90 Minutes

The TLFB and TAS agreed on 35.8% (139/388) of days as alcohol-drinking days and on 46.1% (179/388) of days as abstinent days. There were 10.1% (39/388) of days in which the TLFB reported an alcohol-drinking day and the TAS did not and 8% (31/388) of days in which the TAS reported an alcohol-drinking day and the TLFB did not.

When splitting the groups and conducting a correlation between TAS- and TLFB-reported drinking days, both were positively significantly correlated, but the CM group had a stronger correlation effect (control group: *r*_15_=0.805 and *P*<.001; CM group: *r*_14_=0.913 and *P*<.001).

##### Skyn TAC

Reasons why the TAS did not detect an alcohol event but it was reported in the TLFB could include a large amount of missing data; TAS removal; a low amount of alcohol self-reported; or it being the last day of participation, which may mean that there was not enough time for alcohol to appear in the TAC output. There were other days in which there was no obvious reason why the TAS did not detect alcohol consumption.

Reasons why the TAS reported an alcohol-drinking day but the participant did not self-report alcohol consumption could include a sudden TAC spike, which suggests contact with an alcohol-containing product (eg, aftershave, deodorant, or hand sanitizer); the skin temperature suggesting that the TAS was not being worn at the time of TAC event detection, so the TAS may have been on a table near an alcohol spill or alcohol-containing product; and the TAS appearing to detect an alcohol event, so the self-report was inaccurate. There were other days in which there was no obvious reason why the participant did not self-report alcohol consumption.

**Figure 3:**
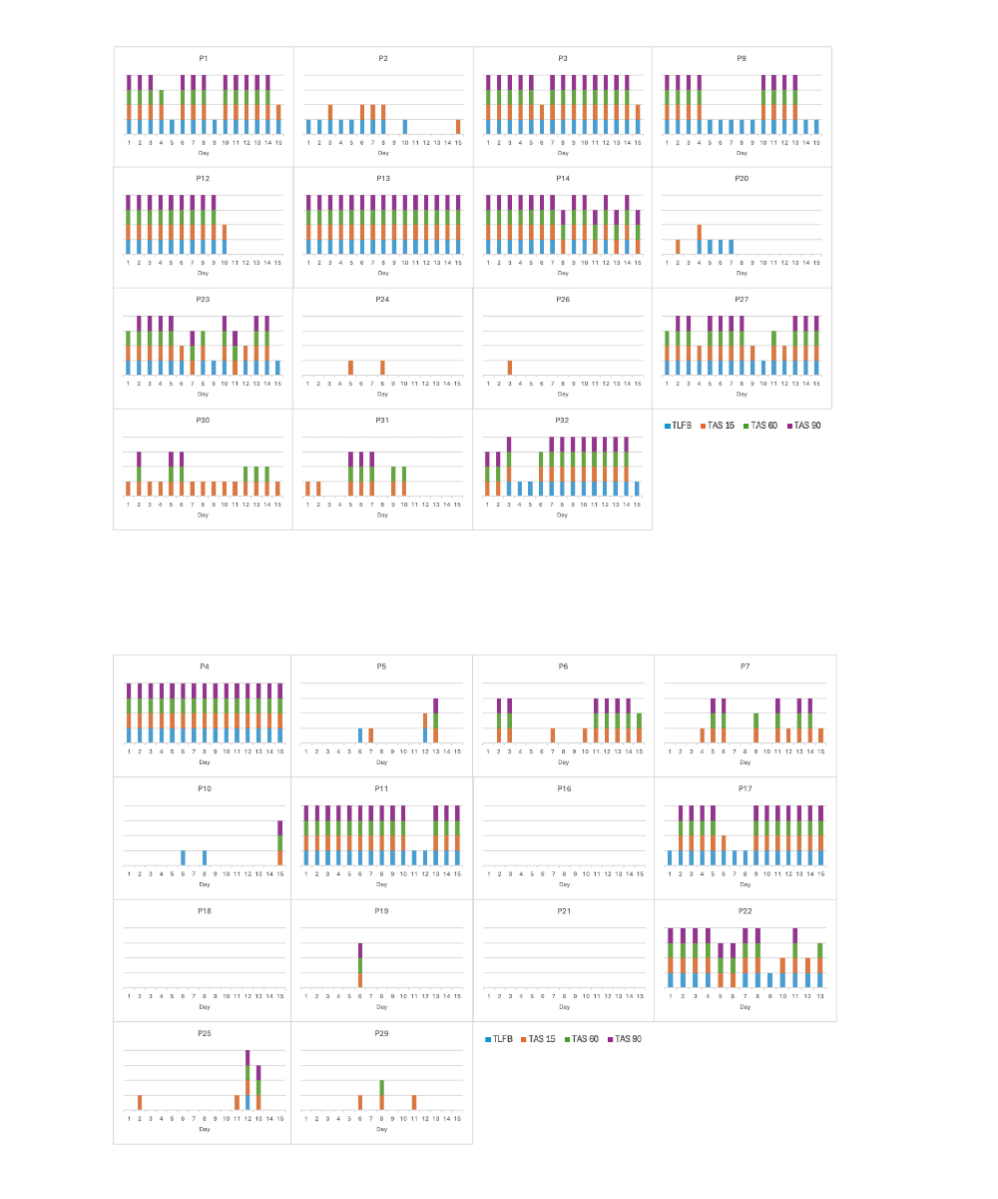
Visual representation of participants’ Timeline Followback (TLFB)– and transdermal alcohol sensor (TAS)–detected alcohol-drinking days for (A) the control group and (B) the contingency management (CM) group. The figure includes 3 different TAS criteria for detecting a drinking day: TAS 15 (transdermal alcohol concentration [TAC]>15 µg/L for >15 min), TAS 60 (TAC>15 µg/L for >60 min), and TAS 90 (TAC>15 µg/L for >90 min).

In Figure 3, a visual presentation of the data for each TAC criteria is provided. It shows TLFB and the TAS criteria of TAS 15 (TAC>15 µg/L for >15 min), TAS 60 (TAC>15 µg/L for >60 min), and TAS 90 (TAC>15 µg/L for >90 min). The presence of the color corresponding to each of these TAC criteria indicates that an alcohol-drinking day was detected.

**Figure 4:**
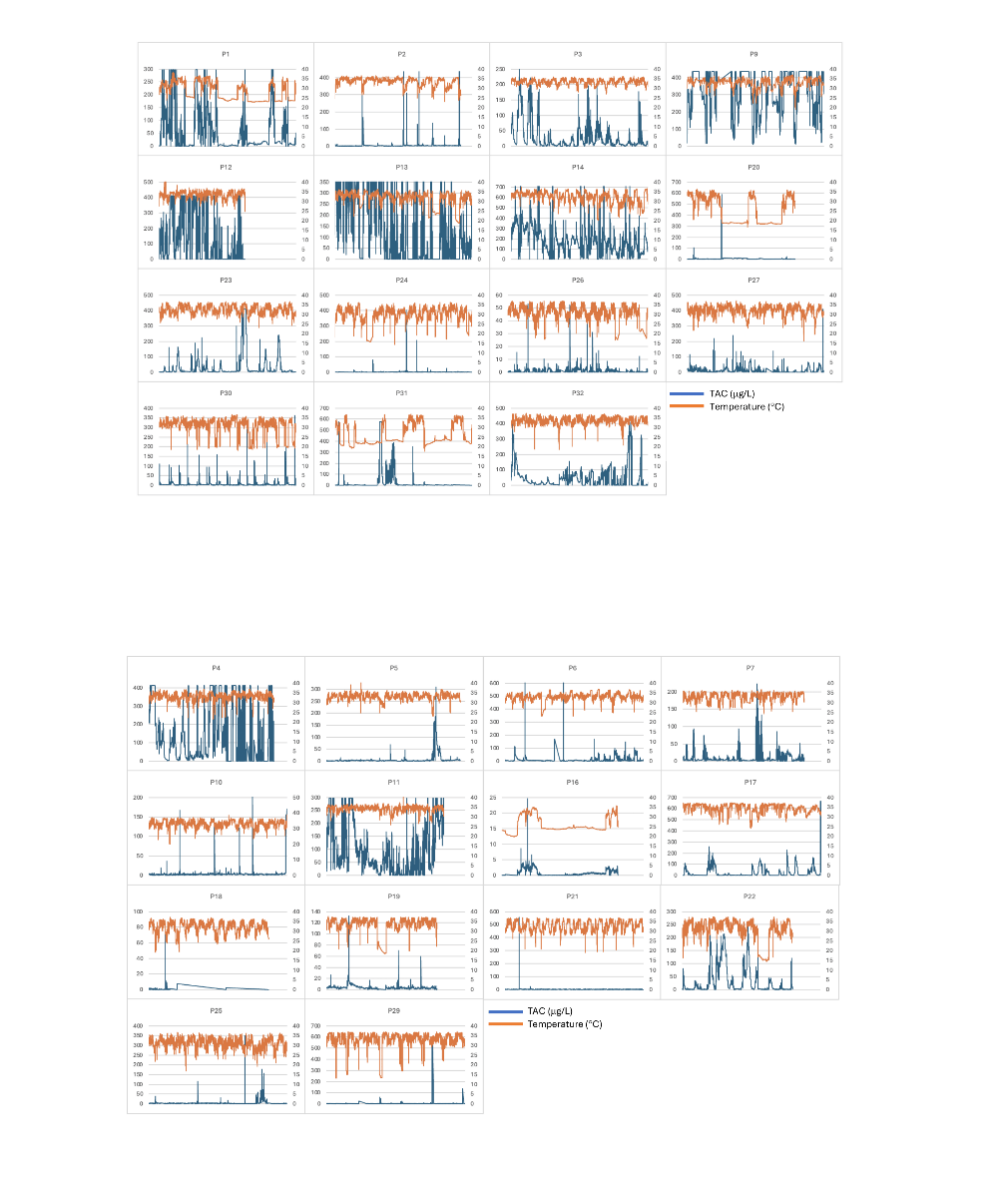
Individual participant Skyn data (excluding withdrawn participants). The primary axis shows the transdermal alcohol concentration (TAC; µg/L) data (blue line), and the secondary axis shows the skin temperature (°C) data (orange line).

Figure 4 shows the data for TAC and the skin temperature of all participants who wore the TAS for the entire study period. There were participants whose TAC (blue line) peaked on separate occasions, suggesting multiple alcohol events, for example, P5 or P17. In contrast, other participant data suggest more frequent alcohol consumption, for example, P9 and P13. The orange lines depict the skin temperature recorded; the skin temperature for P1 and P20 suggests regular, long TAS removal.

##### Sensitivity, Specificity, and Correlations

We calculated the sensitivity, specificity, positive predictive value, negative predictive value, and percentage accuracy in classification for 3 drinking event thresholds: TAS 15 (TAC>15 µg/L for >15 min), TAS 60 (TAC>15 µg/L for >60 min), and TAS 90 (TAC>15 µg/L for >90 min), shown in Table 6.

**Table 6:** Transdermal alcohol concentration (TAC) criteria—sensitivity, specificity, positive predictive value (PPV), negative predictive value (NPV), and percentage accuracy classification (PAC).

Criteria	Sensitivity (%)	Specificity (%)	PPV (%)	NPV (%)	PAC
TAC>15 µg/L for 15 min	92.7	68.29	71.74	91.5	0.80
TAC>15 µg/L for 60 min	82.49	80.5	78.92	83.85	0.81
TAC>15 µg/L for 90 min	78.09	85.31	81.76	82.19	0.82

A Spearman correlation found a significant relationship between TLFB- and Skyn-measured alcohol-drinking days at the TAC>15 µg/L for >15 minutes threshold (*r*_s_=0.695, bias–corrected and accelerated [BCa] 95% CI 0.417-0.883; *P*<.001) [29]. A Spearman correlation between the number of standard alcohol units self-reported and the average TAC values was also found to be significant (*r*_s_=0.770, BCa 95% CI 0.632-0.852; *P*<.001) [29].

A Spearman correlation found a significant relationship between TLFB- and Skyn-measured alcohol-drinking days at the TAC>15 µg/L for >60 minutes threshold (*r*_s_=0.773, BCa 95% CI 0.599-0.861; *P*<.001) [29]. A Spearman correlation between the total number of standard alcohol units self-reported and the TAS-reported drinking days at the TAC>15 µg/L for >60 minutes threshold was also found to be significant (*r*_s_=0.740, BCa 95% CI 0.439-0.891; *P*<.001) [29].

A Spearman correlation found a significant relationship between TLFB- and Skyn-measured alcohol-drinking days at the TAC>15 µg/L for >90 minutes threshold (*r*_s_=0.784, BCa 95% CI 0.625-0.868; *P*<.001) [29]. A Spearman correlation between the total number of standard alcohol units self-reported and the TAS-reported drinking days at the TAC>15 µg/L for >90 minutes threshold was also found to be significant (*r*_s_=0.745, BCa 95% CI 0.460-0.892; *P*<.001) [29].

## Discussion

### Principal Findings

This study aimed to explore the feasibility, strengths, and limitations of using a TAS to monitor alcohol consumption in individuals in treatment for AUD with or without CM to promote abstinence or low-level alcohol consumption. The findings suggest that TAS-delivered CM to encourage alcohol reduction was feasible and acceptable to participants. There was a high correlation between TAS-recorded alcohol-drinking days and self-reported alcohol units, suggesting that the TAS was accurate in measuring the desired behavior. There was also high recruitment, attendance, and compliance of participants. Good rates of meeting attendance and data completeness were achieved. It was feasible to deliver CM using the TAS; however, key features of this process were identified for improvement. This is the first study of TAS-delivered CM in the United Kingdom and the first targeted at alcohol service users for alcohol treatment and reduction.

Participants found the wear of the TAS acceptable and comfortable even for social occasions. They found that it did not interfere with daily activities, and most (19/28, 68%) did not experience any side effects (such as a rash or skin irritation) from the strap. There was a high attendance rate and willingness to participate from those approached. Participants liked the reminder SMS text messages about each meeting, and there were few issues when arranging meetings. Only 3% (1/29) of the participants lost contact with the researcher during participation and did not return the TAS.

However, the answers to the CM survey suggest that this process may need to be improved. Some participants (3/28, 11%) reported it as difficult to understand, and half (7/14, 50%) said that it made no difference or did not help their treatment. These findings suggest that there should be more consideration on how the CM procedure is described and presented to participants at the start. Most (12/14, 86%) said that they did think that CM rewards would help other people who seek treatment for alcohol dependence a little to a lot, with the other 14% (2/14) of the participants saying that it would probably help but only if the patient was motivated.

To deliver the CM, TAS data were downloaded and reviewed at meetings 2 to 7. This was feasible; however, there were issues that arose that would need to be dealt with before scaling up the study size. Considering other options for automated TAC data interpretation will reduce the time-consuming manual data checking, which would only increase the researchers’ time burden if conducted on a larger trial. In addition, creating an automated system could increase consistency and avoid human error.

The recruitment and follow-up rates from this study were the same as or higher than the recruitment and follow-up rates of other TAS feasibility or pilot studies. Previous studies using TASs had sample sizes for analysis of 5 to 13 participants [2,12,34,64]. In total, 2 of these studies used the SCRAM monitor [2,64], and 2 used the BACtrack Skyn [12,34], but 3 [2,34,64] mentioned discomfort with the TAS as key feedback, with Courtney et al [34], who used the BACtrack, having one participant drop out due to discomfort.

Overall, the findings support the feasibility of implementing and delivering CM using a TAS over a 2-week wear period with alcohol service users currently receiving alcohol treatment. The TAS used, BACtrack Skyn, was well liked and rated as comfortable by participants, with little daily interference. The recruitment rates were high and are encouraging for a larger trial. TAS delivery of CM has been shown to be effective in the United States using the SCRAM monitor [1,2,26-31,65], but this pilot shows promise for translation to other TAS brands delivering CM in the United Kingdom, with these findings highlighting aspects to address and improve before scaling to a larger study design. On the basis of these results, future work could explore the possibility of solutions to these challenges and a cost-effectiveness assessment.

In the future, we would also recommend collecting initial data from the participants to determine each participant’s baseline skin temperature. This could be done with the researcher present to ensure correct TAS wear. Some participants were observed by the researcher to be wearing the Skyn during the meeting but the Skyn temperature was reported as <30 °C. Therefore, it may be better to establish a baseline for each participant to then use for their Skyn data for better accuracy at determining wear and removals.

### Strengths and Limitations

The strengths of this study are that it was able to demonstrate the feasible use of the BACtrack Skyn over 2 weeks with individuals currently diagnosed with alcohol dependence and receiving alcohol treatment. Participants wore the TAS in their natural settings and unsupervised, consistent with how it should be worn. While the objective of this study was to assess the feasibility of a larger trial, the data collected as part of this study were able to provide more evidence of how this population wears, uses, and experiences a TAS. There was a high meeting attendance and TAS return rate for this study and no issues with participants being unsure or needing additional training for using the TAS after the baseline training. This study continues to support the use of TASs among the population.

The limitations of this study are similar to those of the design of all the studies conducted as part of this PhD—participants were only recruited if they were willing to wear the TAS from the start of the study period. This means that no participants were recruited who were not willing to attempt wearing the TAS. While this is not a limitation in some considerations as the TAS would be a voluntary treatment option to service users if implemented in services, it does mean that the postwear survey findings may be skewed more positively. Only those who were interested in and willing to wear the TAS had the chance to complete the postwear survey at the end of the 2 weeks. However, participants being willing to wear the TAS for the study does not necessarily mean that they were positive about the technology or that they had a good experience of wearing it; therefore, while we note that the study design does exclude those who are not initially willing to attempt wearing a TAS, this may reflect a truer view of service users who would try wearing the TAS as part of alcohol treatment if TASs were to be implemented in clinical settings.

### Conclusions

To conclude, if planning a future larger trial for TAS delivery of CM, the proposed design changes from this study are (1) changes to how TAC data are checked for CM rewards from a manual to an automatic process, (2) a clearer explanation of the CM procedure, (3) consideration of the times available for participant meetings to improve participant availability and reduce missed appointments, (4) consideration of other TAS brands that have longer data storage, (5) consideration of whether participants should be provided with an iOS device and trained to sync their data or whether participants should have this option with their personal iOS device if using the BACtrack Skyn (which has been deemed feasible by wearers), and (6) consideration of the assessment of each participant’s baseline skin temperature. These points could be assessed via an internal pilot study.

## Appendix

Multimedia Appendix 1 CONSORT (Consolidated Standards of Reporting Trials) checklist.

## Abbreviations

AUD: alcohol use disorder
BCa: bias–corrected and accelerated
CM: contingency management
TAC: transdermal alcohol concentration
TAS: transdermal alcohol sensor
TLFB: timeline followback
